# Body composition parameters, immunonutritional indexes, and surgical outcome of pancreatic cancer patients resected after neoadjuvant therapy: A retrospective, multicenter analysis

**DOI:** 10.3389/fnut.2023.1065294

**Published:** 2023-02-13

**Authors:** Salvatore Paiella, Danila Azzolina, Ilaria Trestini, Giuseppe Malleo, Gennaro Nappo, Claudio Ricci, Carlo Ingaldi, Pier Giuseppe Vacca, Matteo De Pastena, Erica Secchettin, Giulia Zamboni, Laura Maggino, Maria Assunta Corciulo, Marta Sandini, Marco Cereda, Giovanni Capretti, Riccardo Casadei, Claudio Bassi, Giancarlo Mansueto, Dario Gregori, Michele Milella, Alessandro Zerbi, Luca Gianotti, Roberto Salvia

**Affiliations:** ^1^General and Pancreatic Surgery Unit, Pancreas Institute, University of Verona, Verona, Italy; ^2^Department of Environmental and Preventive Science, University of Ferrara, Ferrara, Italy; ^3^Dietetics Services, Hospital Medical Direction, University Hospital Trust of Verona, Verona, Italy; ^4^Department of Biomedical Sciences, Humanitas University, Pieve Emanuele, Italy; ^5^IRCCS Humanitas Research Hospital, Rozzano, Italy; ^6^Pancreatic Surgery Unit, IRCCS Azienda Ospedaliero-Universitaria Di Bologna, Bologna, Italy; ^7^Department of Internal Medicine and Surgery (DIMEC), S. Orsola-Malpighi Hospital, University of Bologna, Bologna, Italy; ^8^Radiology Unit, Pancreas Institute, University of Verona, Verona, Italy; ^9^Unit of Biostatistics, Epidemiology and Public Health, Department of Cardiac, Thoracic, Vascular Sciences and Public Health, University of Padua, Padua, Italy; ^10^General Surgery Unit, University of Siena, Siena, Italy; ^11^School of Medicine and Surgery, University of Milano-Bicocca and HPB Unit, San Gerardo Hospital Monza, Monza, Italy; ^12^Section of Oncology, Department of Medicine, University of Verona Hospital Trust, Verona, Italy

**Keywords:** pancreatic cancer, nutrition–clinical, body composition, postoperative complications, inflammation

## Abstract

**Background and aims:**

Body composition parameters and immunonutritional indexes provide useful information on the nutritional and inflammatory status of patients. We sought to investigate whether they predict the postoperative outcome in patients with pancreatic cancer (PC) who received neoadjuvant therapy (NAT) and then pancreaticoduodenectomy.

**Methods:**

Data from locally advanced PC patients who underwent NAT followed by pancreaticoduodenectomy between January 2012 and December 2019 in four high-volume institutions were collected retrospectively. Only patients with two available CT scans (before and after NAT) and immunonutritional indexes (before surgery) available were included. Body composition was assessed and immunonutritional indexes collected were: VAT, SAT, SMI, SMA, PLR, NLR, LMR, and PNI. The postoperative outcomes evaluated were overall morbidity (any complication occurring), major complications (Clavien-Dindo ≥ 3), and length of stay.

**Results:**

One hundred twenty-one patients met the inclusion criteria and constituted the study population. The median age at the diagnosis was 64 years (IQR16), and the median BMI was 24 kg/m^2^ (IQR 4.1). The median time between the two CT-scan examined was 188 days (IQR 48). Skeletal muscle index (SMI) decreased after NAT, with a median delta of −7.8 cm^2^/m^2^ (*p* < 0.05). Major complications occurred more frequently in patients with a lower pre-NAT SMI (*p* = 0.035) and in those who gained in subcutaneous adipose tissue (SAT) compartment during NAT (*p* = 0.043). Patients with a gain in SMI experienced fewer major postoperative complications (*p* = 0.002). The presence of Low muscle mass after NAT was associated with a longer hospital stay [Beta 5.1, 95%CI (1.5, 8.7), *p* = 0.006]. An increase in SMI from 35 to 40 cm^2^/m^2^ was a protective factor with respect to overall postoperative complications [OR 0.43, 95% (CI 0.21, 0.86), *p* < 0.001]. None of the immunonutritional indexes investigated predicted the postoperative outcome.

**Conclusion:**

Body composition changes during NAT are associated with surgical outcome in PC patients who receive pancreaticoduodenectomy after NAT. An increase in SMI during NAT should be favored to ameliorate the postoperative outcome. Immunonutritional indexes did not show to be capable of predicting the surgical outcome.

## Introduction

Pancreatic cancer (PC) remains a lethal malignancy (1), with a 5-year survival rate of around 30% after surgical resection and multimodal treatment (2). Furthermore, pancreatic surgery's morbidity and mortality rates are still high (3, 4), making the scenario even more problematic.

Pancreatic resections are recognized as one of the most challenging operations due to the magnitude of dissection and resection, the resultant global stress, and the high morbidity rate. Major surgery produces an intense metabolic response and nutritional status changes by activating an inflammatory cascade and releasing stress hormones. Appropriate tissue healing and recovery/maintenance of organ function after such operations necessitate adequate qualitative and quantitative nutritional substrates to be effective. Furthermore, when PC is cephalic, obstructive jaundice is almost invariably present and associated with impaired absorption, nutritional state, and homeostasis (5).

The preoperative identification of patients at risk of malnutrition, and the adoption of nutritional corrective actions, especially in patients receiving systemic therapy before surgery, provides a window of intervention (6) that may mitigate the risk of poor postoperative outcome. Sarcopenia, a progressive decline in skeletal muscle mass, strength, and performance (7), is a direct consequence of impaired nutritional and metabolic status. Based on the patients' populations considered and the cutoff used, the prevalence of sarcopenia in PC patients at diagnosis is variable (8). Research on the association of sarcopenia with surgical outcomes after pancreatic surgery has produced conflicting results (9–11).

Computed tomography (CT) is an accurate tool to quantify whole-body composition (12); moreover, it is routinely used for staging and restaging of PC. Therefore, it is readily available without additional cost, radiation exposure, or inconvenience to the patient. In PC patients, the effects of neoadjuvant therapy (NAT) on body composition have been increasingly investigated, with contrasting results (13–16). In general, lean muscle mass depletion is typical in patients with energetic imbalance and metabolic derangement and may be the driver of a worse surgical outcome.

Chronic systemic inflammation is the theoretical substrate of muscle depletion, sarcopenia, and cachexia (17), and many immunonutritional biochemical parameters have been developed to quantify it (18). Cutoff values of such immunonutritional indexes might serve as a proxy for immunonutritional impairment. Thus, they may help identify fragile patients with an increased pro-inflammatory status, assign patients to appropriate therapies, and even identify early pre-cachexia by offering a multimodal treatment. Among these indexes, the prognostic nutritional index (PNI) (19), the neutrophil-to-lymphocyte ratio (NLR) (20), the platelet-to-lymphocyte ratio (PLR) (21), and the lymphocyte-to-monocyte ratio (LMR) (22) have all been shown to be predictive of surgical or oncological outcome of PC patients.

The current study investigated whether changes in body composition during NAT and multiple preoperative nutritional indexes predict the surgical outcome of locally advanced PC patients who underwent pancreaticoduodenectomy after NAT.

## Methods

### Study design, patient population, and management

The prospective institutional electronic databases of the General and Pancreatic Surgery Unit, Pancreas Institute, University of Verona (Verona, Italy), Milano-Bicocca University at San Gerardo Hospital (Monza, Italy), Pancreatic Surgery Unit, University of Bologna (Bologna, Italy), and of the Pancreatic Surgery Unit of Humanitas University (Milan, Italy) were searched for adult PC patients with NCCN-defined (23) “borderline resectable” or “locally advanced” PC receiving pancreaticoduodenectomy after NAT, between January 2012 and December 2019, of whom two cross-sectional imaging examinations (before and after NAT) and immunonutritional indexes (before surgery) were available.

Regarding individual patient management, each Institution managed each case independently but with a common pathway. Briefly, the chemotherapy choice was left at the oncologist's discretion, and regular multidisciplinary reassessments were made. When the tumor shrunk and/or the Ca 199 levels normalized or at least halved, if radical resection was deemed feasible and the patient was fit, surgery was optioned, and the tumor was ultimately resected. The postoperative care was conducted according to the ERAS recommendations (24).

Given this study's retrospective, observational, and anonymous nature, ethical approval was not required. The study was carried out following the Declaration of Helsinki.

### Body composition assessments and definitions

Weight and height obtained from the patient's chart were recorded by hospital staff. Body mass index (BMI) was obtained by dividing actual weight by height squared (kg/m^2^), and the WHO classification was used for interpretation (25). Skeletal muscle area (SMA), skeletal muscle index (SMI), visceral adipose tissue (VAT), and subcutaneous adipose tissue (SAT) were analyzed from CT images. A single DICOM image was extracted from pre- (at the time of diagnosis/staging) and post-NAT (at restaging before surgery) CT images at the level of the third lumbar vertebra (L3) (26), an area chosen as the best correlate to whole-body composition (27).

DICOM images were then exported to dedicated software, such as CoreSlicer^®^ (28) (Verona and Milan Centers) and ImageJ (29) (Bologna Center). All software, using pre-established Hounsfield unit (HU) thresholds (30), identified and quantified in cm^2^ areas of specific tissues as follows: −29– +150 HU for SM, −190– −30 HU for SAT, and −150– −50 HU for VAT. The skeletal muscle index (SMI) was calculated by normalizing the skeletal muscle area to squared height (in m^2^). Body composition measurements' variation (delta, Δ, calculated as post- minus pre-NAT values) has been calculated.

Acknowledging that the evaluation of muscle quality is mandatory to describe the presence of sarcopenia, and this parameter was not evaluated in the present study, the commonly used term “sarcopenia” has been substituted with “Low muscle mass,” referring to the depletion of lean muscle mass, and the cutoff value proposed by Martin et al. (31) has been adopted.

### Immunonutritional indexes

The immunonutritional indexes were calculated using the laboratory data available at preoperative clinical assessment, typically performed 1–3 weeks before surgery. NLR, PNI, PLR, and LMR were considered continuous variables.

### Surgical outcome

Overall morbidity was the main outcome. It was evaluated considering the rate of postoperative complications (any kind). Secondary metrics for surgical outcome evaluation were:

- Major Complications [defined as Clavien-Dindo (32) grade ≥ 3],
- Length of stay (days).

### Statistical analysis

Descriptive statistics were used to summarize the data from the study variables. Median and interquartile ranges were considered for continuous variables, while, for categorical ones, absolute and relative frequencies were used to synthesize the data. Comparisons of patient characteristics between independent groups were made by calculating the Wilcoxon rank-sum test for continuous variables and the Chi-square test or Fisher's exact test, wherever appropriate, for categorical ones. The effect of SMI on the primary study endpoint was evaluated *via* a logistic regression model accounting for non-linear effects by estimating a restricted cubic spline. The models were adjusted for the characteristics of the patients, such as “sex” and “age.” The SMI cutoff was estimated by identifying the inflection point of the morbidity risk prediction curve. The SMI effects on the morbidity risk are reported in intervals of 5 SMI variations around the inflection point. The effect of SMI on the length of stay has been assessed using the ordinary least squares method with a restricted cubic spline. The Huber-White robust standard error sandwich estimator accounted for the correlation within the repeated pre- and post-measurements. The effect of age on SMI has been assessed using the ordinary least squares method with a linear regression model, adjusted for sex. The 1,000 runs bootstrap 95% confidence intervals have been reported for the prediction plots. The univariable linear regression model results, considering the effect of body composition parameters on the length of stay have been also reported with the estimated effects (Beta) and the 95% confidence intervals.

Analyses were performed with the R system (33) and the rms libraries (34).

## Results

### Patient characteristics

A total of 121 patients met the inclusion criteria and were enrolled in the study. Females and males were almost equally distributed (50.4%/49.6%), the median age at diagnosis was 64 (IQR 16), and the median BMI was 24 kg/m^2^ (IQR 4.1). At diagnosis, 92 (76%) cases were borderline resectable cancer, and the remaining 29 (24%) were locally advanced. The most common chemotherapy regimen was FOLFIRINOX (Fluorouracil-Folinic Acid-Irinotecan-Oxaliplatin, 45.4%), and the median duration of chemotherapy was five cycles (IQR 5). Thirty patients (24.8%) received additional stereotaxic radiation therapy before surgery. At restaging, 63 (52%) and 54 (44.7%) patients had stable and partial/complete responses, respectively. Table 1 reports the general characteristics of the study population, including chemotherapy, surgical, pathologic, and relevant postoperative data.

**Table 1:** Study population's general characteristics (*n* = 121).

**Variable**	**Total, *n* (%)**
Age (years, mean, SD)	61 (10)
Sex (Female)	61 (50.4%)
ASA score III-IV, yes	24 (19.8%)
CACI >4, yes	71 (58.7%)
Diabetes mellitus, yes	27 (22.3%)
NLR (median, IQR)	2.1 (2)
PLR (median, IQR)	140 (59.8)
LMR (median, IQR)	2.6 (2)
PNI (median, IQR)	41 (4.8)
Albumin (g/L, median, IQR)	41 (5.2)
Stage at diagnosis	
Borderline resectable	92 (76)
Locally advanced	29 (24)
Tumor size (mm, mean, SD)
Pre-neoadjuvant therapy	30.6 (8.9)
Post-neoadjuvant therapy	24.3 (9.6)
Neoadjuvant therapy scheme
FOLFIRINOX	55 (45.4)
Gemcitabine/Nab-Paclitaxel	33 (27.3)
Other	23 (27.3)
Chemotherapy duration (cycles, median, IQR)	5 (5)
Time diagnosis to surgery (mo, median, IQR)	6 (5)
Vascular resection, yes	24 (19.8%)
T-status at pathology	
Tx	15 (12.4)
T1	31 (25.6)
T2	58 (47.9)
T3	4 (3.3)
T4	13 (10.7)
N-status at pathology	
N0	47 (38.8)
N1	46 (38)
N2	28 (23.2)
R0 resection, yes	68 (56.2)
Length of stay (days), median (IQR)	11 (9)
Postoperative morbidity (overall), yes	61 (50.4%)
Major complications (Clavien-Dindo ≥ 3), yes	15 (12.4%)

NLR, neutrophil-to-lymphocyte ratio; PLR, platelet-to-lympho-cyte ratio; LMR, lymphocyte-to-monocyte ratio; PNI, prognostic nutritional index.

### Body composition changes after NAT

Table 2 shows the changes in body composition after the completion of NAT. The median time between the two CT scans was 188 days (IQR 48). Before NAT, 36 patients (32.1%) reported low muscle mass, and this percentage increased slightly after NAT (*N* = 41, 33.9%). Muscle components (SMI) or adipose tissue (VAT) components decreased after NAT (all *p* < 0.05). The regression model found that for an increase in age from 54 to 70 years, a decrease in SMI of 5 cm^2^/m^2^ is expected [95%CI (−9.9, −0.2), *p* = 0.04].

**Table 2:** Body composition parameters changes during neoadjuvant therapy.

**Parameter**	**Pre NAT**	**Post NAT**	**Delta**	**95% CI**	***p*-value**
BMI, kg/m^2^	24.0 (4.1)	23.8 (4.0)	−0.14 (1.46)	−1.3, 0.60	0.5
SMA, cm^2^	133 (58)	134 (51)	1.2 (16)	−12, 8.5	0.7
SMI, cm^2^/m^2^	52 (32)	49 (16)	0.34 (13.54)	−13, −2.6	**0.003**
VAT, cm^2^	121 (124)	103 (108)	−8.7 (40.1)	−30, 9.4	**<0.001**
SAT, cm^2^	167 (108)	166 (98)	−8.7 (55.3)	−29, 9.7	0.054

^†^Median (IQR); %; n (%).

NLR, neutrophil-to-lymphocyte ratio; PLR, platelet-to-lymphocyte ratio; LMR, lymphocyte-to-monocyte ratio; PNI, prognostic nutritional index. SMA, skeletal muscle area; SMI, skeletal muscle index; SAT, subcutaneous adipose tissue; VAT, visceral adipose tissue. Bold values indicate statistical significance.

### Body composition changes and surgical outcome

**Figure 1:**
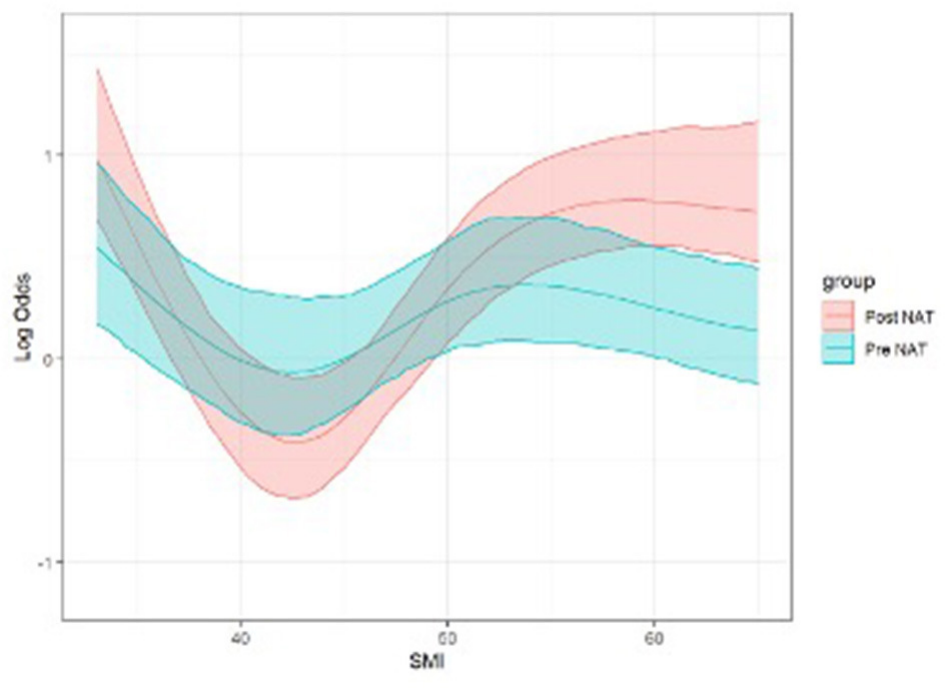
Logistic regression model for postoperative morbidity risk (log-OR) according to SMI (Pre- and Post-NAT) adjusted per gender and age (*p* < 0.001, see text). Both linear (*p* = 0.01) and non-linear (*p* = 0.02) effects are significant. NAT, Neoadjuvant therapy; SMI, skeletal muscle index (cm^2^/m^2^).

Regarding the main study's outcome, general postoperative complications were not associated with changes in the body compartment (Supplementary Table 1). The SMI effects on the morbidity risk are reported in intervals of 5 SMI variations (30–50) around the 42 SMI inflection point. We found that an increase in SMI from 35 to 40 cm^2^/m^2^ reduced the probability of developing any postoperative complications [Log-OR 0.43, 95% CI (0.21, 0.86), *p* < 0.001, Figure 1]. As concern major postoperative complications, they occurred more frequently in patients who had a pre-NAT lower SMI (*p* = 0.035) and a gain in the SAT compartment (*p* = 0.043), and less frequently in patients who had a gain in SMI (*p* = 0.002, Table 3).

**Table 3:** Body composition changes and major postoperative complications.

	**Major Complications (*n* = 15)**	**No major Complications (*n* = 101)**	***p*-value**
SMA, cm^2^ (median, IQR)			
Pre-NT	126 (54)	134 (58)134 (58)	0.8
Post-NT	125 (43)	136 (50)	0.7
Δ	15 (24)	1 (12)	0.066
SMI, cm^2^/m^2^ (median, IQR)			
Pre- NT	44 (10)	53 (35)	**0.035**
Post- NT	49 (16)	50 (12)	0.7
Δ	0 (17)	6 (5)	**0.002**
SAT, cm^2^ (median, IQR)			
Pre- NT	140 (112)	168 (107)	0.7
Post- NT	183 (89)	164 (99)	0.5
Δ	12 (48)	−11 (51)	**0.043**
VAT, cm^2^ (median, IQR)			
Pre- NT	81 (119)	121 (122)	0.5
Post- NT	96 (90)	107 (109)	0.9
Δ	−1 (43)	−10 (39)	0.2
Low muscle mass (32)			
Pre-neoadjuvant therapy	40%	28%	0.432
Post-neoadjuvant therapy	33%	34%	0.961

NLR, neutrophil-to-lymphocyte ratio; PLR, platelet-to-lymphocyte ratio; LMR, lymphocyte-to-monocyte ratio; PNI, prognostic nutritional index. SMA, skeletal muscle area; SMI, skeletal muscle index; SAT, subcutaneous adipose tissue; VAT, visceral adipose tissue. Bold values indicate statistical significance.

When it comes to the length of stay, an increase in VAT (pre- and post-NAT), and the presence of low muscle mass after NAT were associated with a longer stay [Beta 0.03, 95%CI (0.01, 0.05), *p* = 0.010; Beta 0.04, 95%CI (0.02, 0.06), *p* = 0.019; and Beta 5.1, 95%CI (1.5, 8.7), *p* = 0.006, respectively], while an increase in albumin predicted a shorter stay [Beta −0.24, 95%CI (−0.47, −0.02), *p* = 0.039]; Table 4 shows a selection of the variables of the analysis, while Supplementary Table 3 provides the complete list.

**Table 4:** Univariable analysis and length of stay (extracted from Supplementary Table 3).

**Variable**	**Beta**	**95% CI**	***p*-value**
Albumin, g/L	−0.24	−0.47, −0.02	**0.039**
NLR	0.58	−0.17, 1.3	0.13
PNI	−0.07	−0.42, 0.27	0.7
PLR	0.01	−0.01, 0.02	0.4
LMR	−0.50	−1.5, 0.47	0.3
SMA pre-NAT, cm^2^	0.01	−0.03, 0.05	0.6
SMI pre-NAT, cm^2^/m^2^	−0.01	−0.09, 0.07	0.8
VAT pre-NAT, cm^2^	0.03	0.01, 0.05	**0.010**
SAT pre-NAT, cm^2^	0.00	−0.02, 0.02	>0.9
SMA post-NAT, cm^2^	−0.03	−0.07, 0.02	0.2
SMI post-NAT, cm^2^/m^2^	−0.07	−0.17, 0.04	0.2
VAT post-NAT, cm^2^	0.04	0.02, 0.06	**0.019**
SAT post-NAT, cm^2^	0.01	−0.01, 0.03	0.4
Low muscle mass (32) pre-NAT	2.3	−1.5, 6.1	0.2
Low muscle mass (32) post-NAT	5.1	1.5, 8.7	**0.006**

NLR, neutrophil-to-lymphocyte ratio; PLR, platelet-to-lymphocyte ratio; LMR, lymphocyte-to-monocyte ratio; PNI, prognostic nutritional index. SMA, skeletal muscle area; SMI, skeletal muscle index; SAT, subcutaneous adipose tissue; VAT, visceral adipose tissue. Bold values indicate statistical significance.

### Immunonutritional indexes and surgical outcome

None of the immunonutritional indexes proved predictive of a worse postoperative outcome (Table 4, Supplementary Tables 2, 3). In addition, no differences were found when comparing each immunonutritional index in sarcopenic vs. non-sarcopenic patients (data not shown).

## Discussion

Body composition analysis and a careful nutritional assessment are invaluable tools that help identify cancer patients at risk of major postoperative complications. PC patients are not an exception. Typically, they are malnourished and sarcopenic, already at diagnosis. In this study, about one-third of the included patients had a low muscle mass at diagnosis, and this rate remained substantially stable after NAT. The absence of a worsening of sarcopenia, reported by other surgical series (9), may be due to the always increased awareness among patients, caregivers, and healthcare providers of the importance of nutritional status in oncology, especially in PC patients (the majority of the present study patients were enrolled during the last year of the study period).

Regarding the body composition changes that occur during NAT, it was found that both the muscular and the fat compartments were significantly impacted by NAT. These findings have already been reported for PC patients receiving chemotherapy (14, 35–39), demonstrating energetic dyshomeostasis. Therefore, attention must be paid to the body composition changes that occur during NAT in an attempt to maintain patients' body homeostasis, energetic balance, and appropriate metabolism. Radiological reevaluations performed periodically during NAT allow clinicians to achieve it.

When it comes to the study's primary endpoint, while any body composition parameter change did not influence the occurrence of any complications, patients experiencing major complications had a lower pre-NAT SMI value compared with those not facing major complications (*p* < 0.05); additionally, patients having a positive delta SMI (those who gained lean muscle mass) were less likely to experience major postoperative complications. The opposite was true for patients gaining subcutaneous fat tissues after NAT that were more exposed to major complications (all *p* < 0.05). These results align with the fact that the presence of sarcopenia post-NAT predicts a longer length of stay (11). That gaining SAT exposed patients to a greater risk of major postoperative complications is not easily explained because, despite being non-statistically significant, a tendency toward fat tissue loss during NAT was found for both VAT and SAT (Table 1). This finding is likely to be clinically meaningless.

A longer stay was also associated with high VAT values. This finding may be explainable by some factors or events not collected for this study (surgical site infections and, in general, infectious complications), so that patients with a high component of adipose tissue experience a longer hospitalization and, in general, failure to rescue. Instead, an increase in albumin was associated with a shorter length of stay. This recalls previous reports that associated low preoperative albumin levels with a worse postoperative outcome after pancreatic surgery (43–45). However, other studies did not report the same finding (46), and a recent randomized controlled trial demonstrated that the routine correction of preoperative hypoalbuminemia did not lead to a better postoperative outcome (40).

This study presents a novel dynamic model that can identify patients with the greater benefit of gaining lean muscle mass, namely those who move from an SMI of 35 to an SMI of 40 cm^2^/m^2^. This positive change may reduce the odds of experiencing any postoperative complication by about 60%. This aspect points attention to the need to identify patients at high risk of postoperative complications, focusing on those with low SMI who can concretely benefit from a tailored nutritional intervention to reduce the probability of postoperative complications, following a nutritional path, and setting a goal. The other fluctuations of the SMI to values >40 did not show any protective factor vs. major postoperative complications, since at these values of the SMI it is likely that the body can better resist surgical stress and sooner reach homeostasis. However, our results need to be confirmed prospectively.

Among the immunonutritional values, none predicted the postoperative outcome. This result probably reflects the heterogeneity of the study population when it comes to neutrophil and lymphocyte values with respect to having suffered from inflammatory, infectious events before and close to surgery that could have altered these values in the preoperative period (65% of patients had a biliary stent, 25% received multiple endoscopic procedures in the biliary tract, and 15% had had cholangitis).

Of note, we found that a decrease in SMI has to be expected with the increase in age (Supplementary Figure 1). About one-third of 60-year-old patients are sarcopenic (41), and a decrease in lean muscle mass must be expected at a rate of 15% per decade over 70 years (42). Considering that the highest peak of PC incidence occurs between 60 and 80 years, our results underline that nutritional evaluation at the time of diagnosis and during NAT may be fundamental, especially in elderly patients. Pre-habilitation regimens based on exercise (aerobic and resistance activity) and nutritional support focused on maximizing energy and protein intake should focus especially on these subgroups of patients to improve the outcome.

This study has some limitations. First, its retrospective nature does not allow avoiding a selection bias. Second, while the study covers a long period, there was an imbalance toward the last year, when more than half of the cases were recruited. This may have generated a selection and management bias. Third, it cannot be excluded that the enrolled patients could have received nutritional counseling and support during chemotherapy, thus creating another source of bias. Fourth, the assessment of muscle quality (strength or performance) was not done nor feasible, highlighting that muscle mass was evaluated in terms of quantity (low muscle mass) and not quality. Fifth, comparing the results of the present study with the available literature might be inaccurate, as populations are very heterogeneous in terms of disease stages and treatments. Last, the study population is heterogeneous in terms of neoadjuvant treatment and stage disease, and this may impact the results obtained.

## Conclusions

In conclusion, in our experience, the muscle compartment may decrease during NAT, and the delta of variation may provide useful predictive information for the preoperative risk assessment analysis of PC patients undergoing pancreaticoduodenectomy after NAT. For the first time, we identified a subset of patients that may benefit the most from a gain in SMI during NAT, creating a nutritional trajectory to follow and a goal for clinicians to optimize postoperative outcomes. This study failed to prove the ability of the immunonutritional indexes to predict the postoperative outcome; their application may be more appropriate in non-cephalic PC.

## Data availability statement

The original contributions presented in the study are included in the article/Supplementary material, further inquiries can be directed to the corresponding authors.

## Ethics statement

Ethical review and approval was not required for the study on human participants in accordance with the local legislation and institutional requirements. Written informed consent for participation was not required for this study in accordance with the national legislation and the institutional requirements.

## Author contributions

All authors listed have made a substantial, direct, and intellectual contribution to the work and approved it for publication.
