# Corrigendum: Body composition parameters, immunonutritional indexes, and surgical outcome of pancreatic cancer patients resected after neoadjuvant therapy: a retrospective, multicenter analysis

**DOI:** 10.3389/fnut.2023.1212436

**Published:** 2023-05-22

**Authors:** Salvatore Paiella, Danila Azzolina, Ilaria Trestini, Giuseppe Malleo, Gennaro Nappo, Claudio Ricci, Carlo Ingaldi, Pier Giuseppe Vacca, Matteo De Pastena, Erica Secchettin, Giulia Zamboni, Laura Maggino, Maria Assunta Corciulo, Marta Sandini, Marco Cereda, Giovanni Capretti, Riccardo Casadei, Claudio Bassi, Giancarlo Mansueto, Dario Gregori, Michele Milella, Alessandro Zerbi, Luca Gianotti, Roberto Salvia

**Affiliations:** ^1^General and Pancreatic Surgery Unit, Pancreas Institute, University of Verona, Verona, Italy; ^2^Department of Environmental and Preventive Science, University of Ferrara, Ferrara, Italy; ^3^Dietetics Services, Hospital Medical Direction, University Hospital Trust of Verona, Verona, Italy; ^4^Department of Biomedical Sciences, Humanitas University, Pieve Emanuele, Italy; ^5^IRCCS Humanitas Research Hospital, Rozzano, Italy; ^6^Pancreatic Surgery Unit, IRCCS Azienda Ospedaliero-Universitaria Di Bologna, Bologna, Italy; ^7^Department of Internal Medicine and Surgery (DIMEC), S. Orsola-Malpighi Hospital, University of Bologna, Bologna, Italy; ^8^Radiology Unit, Pancreas Institute, University of Verona, Verona, Italy; ^9^Unit of Biostatistics, Epidemiology and Public Health, Department of Cardiac, Thoracic, Vascular Sciences and Public Health, University of Padua, Padua, Italy; ^10^General Surgery Unit, University of Siena, Siena, Italy; ^11^School of Medicine and Surgery, University of Milano-Bicocca and HPB Unit, San Gerardo Hospital Monza, Monza, Italy; ^12^Section of Oncology, Department of Medicine, University of Verona Hospital Trust, Verona, Italy

**Keywords:** pancreatic cancer, nutrition–clinical, body composition, postoperative complications, inflammation

In the published article, there was an error in affiliation number 6. Instead of “Pancreatic Surgery Unit, IRCCS Azienda Ospedaliero-Universitaria Di Bologna, University of Bologna, Bologna, Italy”, it should be “Pancreatic Surgery Unit, IRCCS Azienda Ospedaliero-Universitaria Di Bologna, Bologna, Italy.”

The authors apologize for this error and state that this does not change the scientific conclusions of the article in any way. The original article has been updated.

