# Supplementary material for: Body composition parameters, immunonutritional indexes, and surgical outcome of pancreatic cancer patients resected after neoadjuvant therapy: A retrospective, multicenter analysis
Source: Front Nutr. 2023 Feb 13;10:1065294. doi: 10.3389/fnut.2023.1065294 (PMC9968808; doi:10.3389/fnut.2023.1065294)
Supplement: Supplementary file 1 [file Data_Sheet_1.docx]

**Supplementary Figures**

**Figure S1.** Ordinary least square regression model predicted SMI according to age-adjusted per gender (p = 0.04, see text). Nonlinear restricted cubic spline effect not significant (p=0.22). SMI: skeletal muscle index.

**Supplementary Tables**

**Table S1**. Body composition changes and overall complications.

|  | Complication (any) | |  |
| --- | --- | --- | --- |
|  | Yes (n=61) | No (n=60) | p-value |
| SMA, cm^2^ (median, IQR)  Pre-NT  Post-NT  Δ | 133 (43)  138 (50)  2 (9) | 133 (64)  130 (43)  1 (21) | 0.7  0.7  0.2 |
| SMI, cm^2^/m^2^ (median, IQR)  Pre- NT  Post- NT  Δ | 50 (24)  48 (15)  1 (7) | 55 (45)  50 (19)  0 (34) | 0.2  0.7  0.15 |
| SAT, cm^2^ (median, IQR)  Pre- NT  Post- NT  Δ | 164 (105)  150 (90)  -11 (65) | 174 (108)  179 (114)  -5 (45) | 0.8  0.4  0.5 |
| VAT, cm^2^ (median, IQR)  Pre- NT  Post- NT  Δ | 109 (117)  89 (89)  -9 (38) | 130 (149)  118 (117)  -9 (49) | 0.3  0.4  0.6 |
| Low muscle mass([30](#_ENREF_30))  Pre-neoadjuvant therapy  Post-neoadjuvant therapy | 35%  35% | 25%  33% | 0.4  0.9 |
|  |  |  |  |

SMA: skeletal muscle index; SMI: skeletal muscle index; SAT: subcutaneous adipose tissue;

VAT: visceral adipose tissue.

**Table S2. Univariable analysis and overall morbidity**

| **Variable** | ***Overall morbidity*** | |  | |  |  |
| --- | --- | --- | --- | --- | --- | --- |
|  | *No = 60^†^* | *Yes = 61^†^* | *OR* | *95%CI* | | *p-value* |
| Age | 60 (17) | 66 (15) | 1.00 | 0.99, 1.00 | | **0.007** |
| Sex, female | 53% | 46% | 1.05 | 1.02, 1.10 | | 0.4 |
| ASA Score  0  1  2  3 | 38 (63%)  10 (17%)  7 (12%)  5 (8.3%) | 23 (38%)  14 (23%)  13 (21%)  11 (18%) | 1.62 | 1.15, 2.33 | | **0.007** |
| Diabetes, yes | 25% | 20% | 0.73 | 0.31, 1.73 | | 0.5 |
| CACI ≥ 4 | 53% | 64% | 1.55 | 0.75, 3.24 | | 0.2 |
| Jaundice, yes | 70% | 57% | 0.58 | 0.27, 1.21 | | 0.2 |
| Albumin (g/L) | 41.2 (4.7) | 40.7 (5.5) | 0.98 | 0.93, 1.03 | | 0.4 |
| NLR, median (IQR) | 2.09 (1.65) | 2.39 (2.15) | 1.00 | 0.85, 1.17 | | >0.9 |
| PNI, median (IQR) | 40.9 (4.5) | 41 (4.9) | 1.03 | 0.96, 1.11 | | 0.4 |
| PLR, median (IQR) | 141 (63) | 140 (128) | 0.59 | 0.23, 1.48 | | 0.3 |
| LMR, median (IQR) | 2.69 (2.25) | 2.66 (2.03) | 0.90 | 0.72, 1.09 | | 0.3 |
| Low muscle mass^1^ pre-NAT yes | 35% | 25% | 1.04 | 0.94, 1.13 | | 0.5 |
| Low muscle mass^1^ post-NAT, yes | 35% | 33% | 1.38 | 0.6, 3.12 | | 0.2 |
| Resectability at diagnosis  Borderline  Locally advanced | 45 (75%)  15 (25%) | 48 (79%)  13 (21%) | 0.81 | 0.34, 1.90 | | 0.6 |
| Neoadjuvant, type  Chemotherapy  Chemo- and radiotherapy | 45 (75%)  15 (25%) | 46 (75%)  15 (25%) | 0.98 | 0.43, 2.24 | | >0.9 |
| Type of chemotherapy  FOLFIRINOX  Gemcitabine and Abraxane  Other Gem-based regimens  NiTRO*^‡^* | 32 (53%)  15 (25%)  13 (22%)  0 | 27 (44%)  18 (30%)  15 (25%)  1 (1.6%) | 1.26 | 0.83, 1.94 | | 0.3 |
| Chemotherapy length (cycles) | 5 (7) | 5 (4) | 0.9 | 0.79, 1.00 | | 0.064 |
| Vascular resection (any kind of) | 22% | 18% | 0.8 | 0.32, 1.95 | | 0.6 |
| Intraoperative blood losses, mL | 478 (378) | 400 (400) | 1.00 | 1.00, 1.00 | | 0.8 |
| Surgery duration (minutes) | 430 (166) | 445 (154) | 1.00 | 1.00, 1.00 | | 0.7 |

*^†‡^*Median (IQR); %; n (%)

*^‡^* NiTRO: liposomal irinotecan with 5-fluorouracil, leucovorin and oxaliplatin

NLR: neutrophil-to-lymphocyte ratio; PLR: platelet-to-lymphocyte ratio; LMR: lymphocyte-to-monocyte ratio; PNI: prognostic nutritional index.

**Table S3. Univariable analysis and length of stay**

| **Variable** | **Beta** | **95% CI** | **p-value** |
| --- | --- | --- | --- |
| Age | 0.15 | -0.03, 0.32 | 0.10 |
| Sex, female | -1.3 | -4.8, 2.2 | 0.5 |
| ASA Score | 0.45 | -1.2, 2.1 | 0.6 |
| Diabetes, yes | -1.4 | -5.7, 2.8 | 0.5 |
| CACI ≥ 4 | -0.80 | -4.4, 2.8 | 0.7 |
| Jaundice, yes | -0.80 | -4.5, 2.9 | 0.7 |
| Albumin, g/L | -0.24 | -0.47, -0.02 | **0.039** |
| NLR | 0.58 | -0.17, 1.3 | 0.13 |
| PNI | -0.07 | -0.42, 0.27 | 0.7 |
| PLR | 0.01 | -0.01, 0.02 | 0.4 |
| LMR | -0.50 | -1.5, 0.47 | 0.3 |
| SMA pre-NAT, cm^2^ | 0.01 | -0.03, 0.05 | 0.6 |
| SMI pre-NAT, cm^2^/m^2^ | -0.01 | -0.09, 0.07 | 0.8 |
| VAT pre-NAT, cm^2^ | 0.03 | 0.01, 0.05 | **0.010** |
| SAT pre-NAT, cm^2^ | 0.00 | -0.02, 0.02 | >0.9 |
| SMA post-NAT, cm^2^ | -0.03 | -0.07, 0.02 | 0.2 |
| SMI post-NAT, cm^2^/m^2^ | -0.07 | -0.17, 0.04 | 0.2 |
| VAT post-NAT, cm^2^ | 0.04 | 0.02, 0.06 | **0.019** |
| SAT post-NAT, cm^2^ | 0.01 | -0.01, 0.03 | 0.4 |
| Low muscle mass^1^ pre-NAT, yes | 2.3 | -1.5, 6.1 | 0.2 |
| Low muscle mass^1^ post-NAT, yes | 5.1 | 1.5, 8.7 | **0.006** |
| Resectability (borderline vs. locally advanced) | -1.5 | -5.7, 2.6 | 0.5 |
| Neoadjuvant, type (chemo- vs. chemo- and radiotherapy) | 0.33 | -3.8, 4.4 | 0.9 |
| Type of chemotherapy | 0.20 | -1.9, 2.3 | 0.8 |
| Chemotherapy length (cycles) | -0.44 | -1.0, 0.12 | 0.12 |
| Vascular resection (any kind of) | 1.9 | -2.5, 6.3 | 0.4 |
| Intraoperative blood losses, mL | 0.00 | 0.00, 0.00 | 0.5 |
| Surgery duration, minutes | 0.00 | -0.02, 0.01 | 0.9 |

NLR: neutrophil-to-lymphocyte ratio; PLR: platelet-to-lymphocyte ratio; LMR: lymphocyte-to

-monocyte ratio; PNI: prognostic nutritional index. SMA: skeletal muscle index; SMI: skele-

tal muscle index; SAT: subcutaneous adipose tissue; VAT: visceral adipose tissue.

**Supplementary Material References**

1. Martin L, Birdsell L, Macdonald N, et al. Cancer cachexia in the age of obesity: skeletal muscle depletion is a powerful prognostic factor, independent of body mass index. J Clin Oncol 2013;31:1539-47.
